# Additions to *Diaporthe* (*Sordariomycetes*, *Diaporthaceae*) and *Neptunomyces* (*Dothideomycetes*, *Didymosphaeriaceae*) species associated with *Itea* in Thailand

**DOI:** 10.3897/mycokeys.138.203334

**Published:** 2026-08-07

**Authors:** Jutamart Monkai, Rungtiwa Phookamsak, Surapong Khuna, Darbhe Jayarama Bhat, Toe Swe Zin Ei, Jianchu Xu, Sinang Hongsanan, Saisamorn Lumyong

**Affiliations:** 1 Office of Research Administration, Chiang Mai University, Chiang Mai 50200, Thailand Shenzhen Key Laboratory of Microbial Genetic Engineering, College of Life Sciences and Oceanography, Shenzhen University Shenzhen China; 2 Department of Biology, Faculty of Science, Chiang Mai University, Chiang Mai 50200, Thailand Honghe Center for Mountain Futures, Kunming Institute of Botany, Chinese Academy of Sciences Honghe China; 3 Centre for Mountain Futures (CMF), Kunming Institute of Botany, Kunming 650201, Yunnan, China Centre for Mountain Futures (CMF), Kunming Institute of Botany Kunming China; 4 Honghe Center for Mountain Futures, Kunming Institute of Botany, Chinese Academy of Sciences, Honghe 654400, China Department of Economic Plants and Biotechnology, Yunnan Key Laboratory for Wild Plant Resources, Kunming Institute of Botany, Chinese Academy of Sciences Kunming China; 5 Department of Economic Plants and Biotechnology, Yunnan Key Laboratory for Wild Plant Resources, Kunming Institute of Botany, Chinese Academy of Sciences, Kunming 650201, Yunnan, China Department of Botany and Microbiology, College of Science, King Saud University Riyadh Saudi Arabia; 6 Shenzhen Key Laboratory of Microbial Genetic Engineering, College of Life Sciences and Oceanography, Shenzhen University, Shenzhen 518060, China Academy of Science, The Royal Society of Thailand Bangkok Thailand; 7 Department of Botany and Microbiology, College of Science, King Saud University, Riyadh 11451, Saudi Arabia Department of Biology, Faculty of Science, Chiang Mai University Chiang Mai Thailand; 8 Biology Division, Vishnugupta Vishwavidyapeetam, Ashoke, Gokarna 581326, India Office of Research Administration, Chiang Mai University Chiang Mai Thailand; 9 The Center for International Forestry Research and World Agroforestry (CIFOR-ICRAF) China Program, World Agroforestry (ICRAF), Kunming 650201, Yunnan, China Center of Excellence in Microbial Diversity and Sustainable Utilization, Chiang Mai University Chiang Mai Thailand; 10 Center of Excellence in Microbial Diversity and Sustainable Utilization, Chiang Mai University, Chiang Mai 50200, Thailand Biology Division, Vishnugupta Vishwavidyapeetam Gokarna India; 11 Academy of Science, The Royal Society of Thailand, Bangkok 10300, Thailand The Center for International Forestry Research and World Agroforestry (CIFOR-ICRAF) China Program, World Agroforestry (ICRAF) Kunming China

**Keywords:** Coelomycetous asexual morph, *Diaporthe siamensis* species complex, endophytic fungi, *Itea*-inhabiting fungi, novel species, polyphasic taxonomy, species identification

## Abstract

*Itea* is an economically and medicinally important plant, widely distributed across China, Japan and Southeast Asia, noted for producing rare sugars. Despite this, endophytic fungi associated with this plant remain poorly studied. This study focuses on the taxonomy and phylogeny of fungi isolated from healthy tissues of *Itea japonica* and *I. riparia* in Northern Thailand. By incorporating morphology, multiloci phylogenetic analyses and nucleotide polymorphism comparison, we establish two novel species, *Diaporthe iteae* (*Diaporthaceae*, *Sordariomycetes*) and *Neptunomyces iteae* (*Didymosphaeriaceae*, *Dothideomycetes*) as well as report a new host and a new geographical record for *Diaporthe coracoralinae*. *Diaporthe iteae***sp. nov**. was collected from *I. japonica*, and is recognized within the *Diaporthe siamensis* species complex (section *Sojae*). In addition, *D. coracoralinae* is reported on *I. japonica* in Thailand for the first time, occupying a position within section *Sojae* with an undetermined species complex. *Neptunomyces iteae***sp. nov**. was collected from *I. riparia* and is classified within the family *Didymosphaeriaceae*. Updated phylogenetic trees and comprehensive morphological comparisons with closely related taxa are provided and discussed for better resolution of species delimitation. This study enhances our understanding of fungal diversity on *Itea*, and provides a taxonomic foundation for future biodiversity, ecological and applied studies.

## Introduction

*Diaporthe* (*Diaporthaceae*, *Sordariomycetes*) is an important genus of coelomycetous *Ascomycota* established by Nitschke (1870) with the type species, *D. eres*. *Diaporthe* commonly exists as endophytes, pathogens, and saprobes and is associated with diverse hosts and has a broad geographic distribution and their relationships within the *Diaporthales* still warrant further investigations (Rossman et al. 2007; Udayanga et al. 2011; Gomes et al. 2013; Senanayake et al. 2017, 2018; Perera et al. 2018; Huang et al. 2021; Ferro et al. 2024; Gomzhina and Gasich 2026). Previously, asexual morph in *Diaporthe* was treated as *Phomopsis*. According to the “one fungus one name” nomenclature (Rossman et al. 2015), *Diaporthe* has priority over the synonym *Phomopsis*. The asexual morph of this genus is characterized by ostiolate conidiomata, cylindrical phialides, aseptate, hyaline and three types of conidia (i.e. alpha, gamma, and beta) (Udayanga et al. 2011; Gomes et al. 2013), while the sexual morph is characterized by pseudostromatic ascomata with perithecial necks, unitunicate asci, septate or aseptate, hyaline, ellipsoid to fusiform ascospores, occasionally with appendages (Wehmeyer 1933; Senanayake et al. 2018). The classification of *Diaporthe* species was primarily based on host specificity and morphology (Uecker 1988; Udayanga et al. 2011). However, the main morphological characteristics such as conidiogenous cells and conidia were shared by species that are not closely related phylogenetically (Dissanayake et al. 2017; Gao et al. 2017; Norphanphoun et al. 2022). Therefore, polyphasic taxonomy incorporating morphology, multiloci phylogeny and ecological data has been extensively accepted for species boundaries (Udayanga et al. 2012; Perera et al. 2018; Dissanayake et al. 2020; Hongsanan et al. 2023; Monkai et al. 2023; Ferro et al. 2024; Aumentado et al. 2026). At present, more than 1300 species epithets of *Diaporthe* are listed in Index Fungorum (2026) with a significant increase of species number in recent years (Bhunjun et al. 2022; Aumentado et al. 2026).

*Neptunomyces* (*Didymosphaeriaceae*, *Dothideomycetes*) was proposed by Gonçalves et al. (2019) with *N. aureus* designated as the type species. *Neptunomyces* is a member of *Didymosphaeriaceae*, which is a large family in *Pleosporales* (Gonçalves et al. 2019; Hongsanan et al. 2020). *Didymosphaeriaceae* exhibit a remarkably wide range of hosts and habitats with worldwide distribution, predominantly existing as saprobes or as endophytes and pathogens (Ariyawansa et al. 2014a, 2014b; Yuan et al. 2020; Tennakoon et al. 2022; Ren et al. 2022; Htet et al. 2024; Wanasinghe et al. 2025). Morphologically, the sexual morph of *Didymosphaeriaceae* is characterized by ostiolate ascomata, trabeculate pseudoparaphyses, uni-septate, brown ascospores, where its asexual morphs may be coelomycetes and hyphomycetes (Ariyawansa et al. 2014a, 2014b; Wanasinghe et al. 2016; Tennakoon et al. 2022, 2025). Recently, molecular phylogeny coupled with morphology has been applied to resolve the boundaries of taxa in *Didymosphaeriaceae* (Ren et al. 2022; Tennakoon et al. 2022, 2025; Htet et al. 2024; Wanasinghe et al. 2024, 2025). *Neptunomyces* can be distinguished from other genera in *Didymosphaeriaceae* by producing coelomycetous, aseptate, hyaline to golden yellow conidia with round apices and also forms a monophyletic clade within this family (Gonçalves et al. 2019; Tennakoon et al. 2025). Currently, *Neptunomyces* comprises seven species according to Index Fungorum (2026).

Endophytic fungi have recently been redefined and they are known to constitute symbiotic interaction with host plants and are recognized as prolific sources of bioactive substances, which modulate the accumulation of secondary metabolites (Palwe et al. 2021; Reyes et al. 2021; Yu et al. 2024; Liao et al. 2025). These fungi foster plant growth and increase host resistance to diseases and environmental pressures (Yu et al. 2024; Xu et al. 2026). *Itea* species are particularly valued for their nutritional and therapeutic qualities, which are largely attributed to their ability to accumulate rare sugars and important bioactive compounds (Luo et al. 2014; Zeng et al. 2015; Lang et al. 2022; Feng et al. 2024). Studying the fungal communities within *Itea* is crucial for searching for novel sources of these compounds. Despite the widespread distribution of *Itea* throughout East Asia (Song and Zhang 2024; POWO 2026), research on endophytic fungi associated with this plant remains limited. To address this gap, we carried out taxonomic studies to characterize endophytic isolates from *Itea riparia* and *I. japonica* in Thailand (Monkai et al. 2025, 2026). During our observations, we found four coelomycetous strains that produced distinct conidial exudates in culture. The objectives of this study are to identify these taxa based on multiloci phylogeny, nucleotide sequence comparisons and morphology, and clarify taxonomic positions of these and related taxa within the genus and family.

## Materials and methods

### Fungal Collection and Isolation

Fresh plant samples of *Itea japonica* and *I. riparia* were collected from Hang Dong District and Mae Rim District, Chiang Mai Province, Thailand. Endophytic fungi were isolated from healthy tissues using the surface sterilization method described in a previous study (Monkai et al. 2025). The plant segments (approximately 5 mm^2^) were excised and surface-sterilized by rinsing in sterile distilled water for 1 min, immersing in 70% ethanol for 30 s, followed by 2% NaOCl for 30 s, then air-dried and placed onto potato dextrose agar (PDA) plates. The isolated plates were incubated at 25 °C for one to two days, and then the single-hyphal tips were transferred to fresh PDA for purification and further morphological observation. Pure cultures were deposited and permanently preserved in a metabolically inactive state at the Mae Fah Luang University Culture Collection (**MFLUCC**), Mae Fah Luang University, Chiang Rai Province, Thailand and the Culture Collection of Microbial Shenzhen University (**MBSZU**), Shenzhen University, China. Dried cultures were prepared for fungarium specimens (Monkai et al. 2025) and deposited in the herbarium of Mae Fah Luang University (**MFLU**), Mae Fah Luang University, Chiang Rai Province, Thailand.

### Morphological Characterization

After incubation at 25 °C for one to two months, fungal PDA cultures were examined for sporulation, colony features, pigment production and growth rate. Macro-morphology was examined using a stereo microscope (Nikon SMZ800N, Tokyo, Japan). Micro-morphological characteristics including conidiomata, conidiomatal wall, ostioles, conidiophores, conidiogenous cells and conidia were observed using a compound microscope equipped with Nikon DS-Ri2 camera (Nikon Eclipse Ni U, Tokyo, Japan). Measurements were conducted by the Tarosoft (R) Image Frame Work program with 10 measurements taken for conidiomata, conidiomatal wall, ostioles, conidiophores, conidiogenous cells and 30 measurements for conidia. The morphological description was prepared coupled with photo plates, which were organized by Adobe Photoshop version 21.2.4 (Adobe Systems, San Jose, CA, USA). The newly introduced taxa were registered to obtain Index Fungorum numbers (Index Fungorum 2026).

### DNA extraction, amplification, and sequencing

The fungal mycelium (grown on PDA at 25 °C for one week) was processed for DNA extraction using an E.Z.N.A^®^ Tissue DNA Kit (Omega, USA). Polymerase chain reaction (PCR) was used to amplify the following genomic regions including the internal transcribed spacer region of ribosomal DNA (ITS), the partial 28S large subunit nuclear ribosomal DNA (LSU), 18S small subunit rDNA (SSU), the translation elongation factor 1-α (*tef1-α*), beta-tubulin (*tub2*), calmodulin (*cal*), and histone H3 (*his3*). The specific primers and PCR thermal cycling programs for each gene region are listed in Table 1. PCR amplifications were conducted in a total volume of 20 μl, which contained 7 μl of deionized water, 10 μl of 2× Phanta Max Master Mix (Dye Plus) (Vazyme, China), 1 μl of genomic DNA, and 1 μl of each forward and reverse primer. The PCR products were purified using the E.Z.N.A^®^ Gel Extraction Kit (Omega, USA), and assessed using 1% agarose gel electrophoresis and a Nanodrop 2000 Spectrophotometer (Thermo Scientific, USA). The DNA sequencing of purified PCR samples was performed by BGI-Shenzhen Company (Shenzhen, Guangdong, China).

**Table 1.** The specific primer and PCR thermal cycling program of each gene region used in this study.

**Gene region**	**Primer**	**References**	**PCR thermal cycling program**
ITS	ITS4/ITS5	White et al. (1990)	An initial denaturation step of 5 min at 94 °C followed by 35 cycles of 30 s at 94 °C, 50 s at 52 °C and 1 min at 72 °C, and a final elongation step of 7 min at 72 °C
LSU	LR0R/LR5	Vilgalys and Hester (1990)	
SSU	NS1/NS4	White et al. (1990)	
*tef1-α*	EF1-983F/EF1-2218R	Rehner (2001)	An initial denaturation step of 5 min at 94 °C followed by 35 cycles of 30 s at 94 °C, 50 s at 54 °C and 1 min at 72 °C, and a final elongation step of 7 min at 72 °C
	EF1-728F/EF1-986R	Carbone and Kohn (1999)	
*tub2*	Bt2a/Bt2b	Glass and Donaldson (1995)	
*cal*	CAL228F/CAL737R	Carbone and Kohn 1999	
*his3*	CYLH3F/H3-1b	Glass and Donaldson (1995); Crous et al. (2004)	

### Phylogenetic analyses

The consensus sequence was processed using SeqMan v. 7.0.0 (DNASTAR, Madison, WI). The obtained nucleotide sequence was initially checked by the NCBI nucleotide BLAST search (www.ncbi.nlm.nih.gov/blast/; accessed on 1 March 2026) to identify the closest matches. The reference nucleotide sequences were obtained based on the results of the BLAST search and recently published data of *Diaporthe* section *Sojae* (Dissanayake et al. 2024; Ferro et al. 2024; Aumentado et al. 2026) and *Neptunomyces* (Yasanthika et al. 2024; Chen et al. 2025; Tennakoon et al. 2025) (Tables 2, 3). The single gene sequences were aligned using the MAFFT v7.307 online version (Katoh et al. 2017; https://mafft.cbrc.jp/alignment/server/, accessed on 1 October 2025) and corrected manually using BioEdit version 7.0.5.3 (Hall 1999). The aligned sequence data of ITS, *tef1-α*, *tub2*, *cal* and *his3* were concatenated for phylogenetic analysis of *Diaporthe* section *Sojae*. While the alignment of ITS, LSU, SSU and *tef1-α* was concatenated for phylogenetic analysis of *Didymosphaeriaceae*. Phylogenetic trees were constructed using Maximum likelihood (ML) and Bayesian inference (BI) analyses via the CIPRES Science Gateway platform V3.3 (Miller et al. 2010). For ML analysis, ML node support was estimated with 1,000 bootstrap (BS) replicates and the GTRGAMMA model using RAxML-HPC2 on XSEDE (v.8.2.12) (Stamatakis 2014). Model selection of BI analysis was implemented by MrModelTest v.2.3 based on the Akaike Information Criterion (AIC) (Nylander 2004). The results of the best-fit model for each gene region are shown in Table 4. For BI analysis, Bayesian posterior probability (BYPP) was evaluated with six simultaneous Markov chains and sampling tree every 1000^th^ generation using MrBayes v.3.2.6 (Ronquist et al. 2012). The burn in phase of initial 25% generated trees were discarded, while the remaining trees were applied to calculate posterior probabilities (PPs), when the average standard deviation of split frequencies dropped below 0.01 (Rambaut et al. 2018). The phylograms were visualized and refined in FigTree v1.4.0 (Rambaut and Drummond 2012) and Adobe Illustrator version 24.3 (Adobe Systems, San Jose, CA, the USA). The information on newly described strains was submitted for acquiring GenBank accession numbers. The concatenated alignments and phylogenetic trees are available in Figshare (https://doi.org/10.6084/m9.figshare.33091040).

**Table 2.** GenBank accession numbers of *Diaporthe* section *Sojae* used in multiloci phylogenetic analyses.

**Species name**	**Strain number**	**ITS**	***tef1-α***	***tub2***	***cal***	***his3***
***Diaporthe ambigua***	**CBS 114015**	**MH862953**	**KC343736**	**KC343978**	**KC343252**	**KC343494**
*D. ambigua*	CBS 117167	KC343011	KC343737	KC343979	KC343253	KC343495
***D. biguttulata***	**CFCC 52584**	**MH121519**	**MH121561**	**MH121598**	**MH121437**	**MH121477**
*D. biguttulata*	CFCC 52585	MH121520	MH121562	MH121599	MH121438	MH121478
***D. brasiliensis***	**CBS 133183**	**KC343042**	**KC343768**	**KC344010**	**KC343284**	**KC343526**
*D. brasiliensis*	LGMF926	KC343043	KC343769	KC344011	KC343285	KC343527
***D. caatingaensis***	**URM7485**	**KY085927**	**KY115604**	**KY115601**	**KY115598**	**KY115605**
*D. caatingaensis*	URM7484	KY085928	N/A	KY115602	KY115599	KY115606
***D. caryae***	**CFCC 52563**	MH121498	MH121540	MH121580	MH121422	MH121458
*D. caryae*	CFCC 52564	MH121499	MH121541	MH121581	MH121423	MH121459
***D. cerradensis***	**CMRP4331**	**MN173198**	**MT311685**	**MW751671**	**MW751655**	**MW751663**
*D. cerradensis*	CMRP4324	MN173195	MT311681	MW751667	MW751651	MW751659
***D. chiangraiensis***	**MFLUCC 17-1669**	**MF190119**	**MF377598**	**N/A**	**N/A**	**N/A**
*D. chiangraiensis*	MFLUCC 17-1670	MF190118	MF377599	N/A	N/A	N/A
***D. cinnamomi***	**CFCC 52569**	**MH121504**	**MH121546**	**MH121586**	**N/A**	**MH121464**
*D. cinnamomi*	CFCC 52570	MH121505	MH121547	MH121587	N/A	MH121465
***D. citriasiana***	**ZJUD30**	**JQ954645**	**JQ954663**	**KC357459**	**KC357491**	**N/A**
*D. citriasiana*	ZJUD81	KJ490616	KJ490495	KJ490437	N/A	KJ490558
***D. coracoralinae***	**URM 8912**	**PP192078**	**PP430449**	**PP402241**	**PP408214**	**PP421133**
*D. coracoralinae*	IR7	PP192079	N/A	PP402242	PP408215	PP421134
*D. coracoralinae*	IR6A	PP192077	N/A	PP402239	PP408213	N/A
*D. coracoralinae*	AR11B	N/A	N/A	PP402236	PP408212	PP421132
*D. coracoralinae*	MFLUCC 26-0215	PZ458814	PZ501085	PZ501081	PZ501083	PZ501089
***D. cyatheae***	**YMJ-1364**	**JX570889**	**KC465406**	**KC465403**	**KC465410**	**N/A**
***D. discoidispora***	**ZJUD89**	**KJ490624**	**KJ490503**	**KJ490445**	**N/A**	**KJ490566**
***D. eleutherrhenae***	**01**	**OK017069**	**OK017070**	**OK017071**	**N/A**	**N/A**
*D. eleutherrhenae*	02	OK648457	OK648458	OK648459	N/A	N/A
***D. eres* (*D. alnea*)**	**CBS 146.46**	**KC343008**	**KC343734**	**KC343976**	**KC343250**	**KC343492**
***D. eres* (*D. nobilis*)**	**CBS 587.79**	**KC343153**	**KC343879**	**KC344121**	**KC343395**	**KC343637**
*D. fici-septicae*	NCYUCC 19-0108	MW114349	MW192212	MW148269	N/A	N/A
***D. fici-septicae***	**MFLUCC 20-0178**	**MW114348**	**MW192211**	**MW148268**	**N/A**	**N/A**
***D. foliorum***	**CMRP 1321**	**MT576688**	**MT584310**	**MT584327**	**MT584341**	**MT584338**
*D. foliorum*	CMRP 1320	MT576671	MT584309	MT584328	MT584342	MT584340
***D. ganjae***	**CBS 180.91**	**KC343112**	**KC343838**	**KC344080**	**KC343354**	**KC343596**
*D. ganjae*	PSCG489	MK626955	MK654897	MK691287	MK691202	MK726204
***D. goulteri***	**BRIP 55657a**	**KJ197290**	**KJ197252**	**KJ197270**	**N/A**	**N/A**
***D. infecunda***	**CBS 133812**	**KC343126**	**KC343852**	**KC344094**	**KC343368**	**KC343610**
*D. infecunda*	LGMF908	KC343127	KC343853	KC344095	KC343369	KC343611
***D. iteae***	**MFLUCC 26-0214**	**PZ458813**	**PZ501084**	**PZ501080**	**PZ501082**	**PZ501088**
***D. juglandigena***	**GUCC 422.16**	**OP581229**	**OP688534**	**OP688559**	**N/A**	**N/A**
*D. juglandigena*	GUCC 422.161	OP581230	OP688535	OP688560	N/A	N/A
***D. kyushuensis***	**STE-U2675**	**AF230749**	**N/A**	**N/A**	**N/A**	**N/A**
*D. kyushuensis*	ch-D-1	AB302250	N/A	N/A	N/A	N/A
***D. leucospermi***	**CBS 111980**	**N/A**	**KY435632**	**KY435673**	**KY435663**	**KY435653**
*D. leucospermi*	CAA483	KY435635	KY435625	KY435666	KY435656	KY435645
***D. longispora***	**CBS 194.36**	**KC343135**	**KC343861**	**KC344103**	**KC343377**	**KC343619**
***D. mangroviorum***	**MFLUCC 24-0445**	**PQ699353**	**PV769004**	**PV753771**	**PV753687**	**PV752668**
*D. mangroviorum*	MFLUCC 24-0446	PQ699362	PV769015	PV753782	PV753689	PV752682
***D. mayteni***	**CBS 133185**	**KC343139**	**KC343865**	**KC344107**	**KC343381**	**KC343623**
***D. middletonii***	**BRIP 54884e**	**KJ197286**	**KJ197248**	**KJ197266**	**N/A**	**N/A**
*D. middletonii*	BRIP 57329	KJ197285	KJ197247	KJ197265	N/A	N/A
***D. minusculata***	**CGMCC3.20098**	**MT385957**	**MT424692**	**MT424712**	**MW022475**	**MW022499**
*D. minusculata*	GZCC 19-0345	MT797184	MT793027	MT793038	MW022476	MW022500
***D. orixae***	**KUNCC 21-10714**	**OK283041**	**N/A**	**N/A**	**OK484485**	**OK484486**
*D. orixae*	GZCC 21-1085	OL889852	OL944724	OL944726	N/A	N/A
***D. oxe***	**CBS 133186**	**KC343164**	**KC343890**	**KC344132**	**KC343406**	**KC343648**
*D. oxe*	CBS 133187	KC343165	KC343891	KC344133	KC343407	KC343649
***D. pachirae***	**CDA 728**	**MG559537**	**MG559539**	**MG559541**	**MG559535**	**N/A**
*D. pachirae*	CDA 730	MG559538	MG559540	MG559542	MG559536	N/A
***D. paranensis***	**CBS 133184**	**KC343171**	**KC343897**	**KC344139**	**KC343413**	**KC343655**
*D. paranensis*	LMICRO417	KY461115	KY461116	N/A	N/A	N/A
***D. passiflorae***	**CBS 132527**	**JX069860**	**N/A**	**N/A**	**N/A**	**KY435654**
*D. passiflorae*	DJY16A1-5	MH595929	MH621353	MH621349	N/A	N/A
***D. quercicola***	**CSUFTCC104**	**ON076567**	**ON081659**	**N/A**	**ON081670**	**ON081667**
*D. quercicola*	CSUFTCC105	ON076568	ON081660	N/A	ON081671	ON081668
***D. raonikayaporum***	**CBS 133182**	**KC343188**	**KC343914**	**KC344156**	**KC343430**	**KC343672**
***D. raonikayaporum* (*D. neoraonikayaporum*)**	**MFLUCC 14-1136**	**KU712449**	**KU749369**	**KU743988**	**KU749356**	**N/A**
***D. rhizophoracearum***	**MFLUCC 24-0449**	**PQ699356**	**PV769008**	**PV753774**	**PV753681**	**PV752671**
*D. rhizophoracearum*	MFLUCC 24-0450	PQ699357	PV769009	PV753775	PV753682	PV752672
***D. sclerotioides***	**CBS 296.67**	**MH858974**	**KC343919**	**KC344161**	**KC343435**	**KC343677**
*D. sclerotioides*	CBS 710.76	KC343194	KC343920	KC344162	KC343436	KC343678
*D. sclerotioides* (*D. columnaris*)	AR3612	AF439625	N/A	N/A	N/A	N/A
*D. sclerotioides* (*D. columnaris*)	ICMP 16836	KC145883	KC145999	N/A	N/A	N/A
***D. serafiniae***	**BRIP 55665a**	**KJ197274**	**KJ197236**	**KJ197254**	**N/A**	**N/A**
*D. serafiniae*	BRIP 54136	KJ197273	KJ197235	KJ197253	N/A	N/A
*D. siamensis*	MFLUCC 12-0300	KT459417	KT459451	KT459435	KT459467	N/A
***D. siamensis***	**MFLUCC 10-0573a**	**JQ619879**	**JX275393**	**JX275429**	**JX197423**	**N/A**
***D. submersa***	**GZCC 19-0129**	**OP056717**	**OP150556**	**OP150633**	**OP150710**	**OP150786**
*D. submersa*	GZCC 22-0007	OP056718	OP150557	OP150634	OP150711	OP150787
***D. tulliensis***	**BRIP 62248a**	**KR936130**	**KR936133**	**KR936132**	**N/A**	**N/A**
*D. tulliensis*	RST-1	MN911382	MT121969	N/A	N/A	N/A
***D. yunnanensis***	**CGMCC3.18289**	**KX986796**	**KX999188**	**KX999228**	**KX999290**	**KX999267**
*D. yunnanensis*	LC8107	KY491542	KY491552	KY491562	KY491572	N/A

Notes: Sequences shaded in blue were isolated in this study. Sequences in bold are the ex-type strains. Unavailable sequences are indicated as “N/A”.

**Table 3.** GenBank accession numbers of taxa in *Didymosphaeriaceae* used in multiloci phylogenetic analyses.

**Species name**	**Strain number**	**ITS**	**LSU**	**SSU**	***tef1-α***
***Alloconiothyrium aptrootii***	**CBS 980.95**	**JX496121**	**JX496234**	**N/A**	**N/A**
*A. aptrootii*	CBS 981.95	JX496122	JX496235	N/A	N/A
*A. encephalarti*	CPC: 35980	MN562102	MN567610	N/A	N/A
*Austropleospora archidendri*	MFLUCC 17-2429	MK347757	MK347974	MK347863	MK360044
*A. archidendri*	MFLU 22-0042	OP058964	OP059055	OP059006	OP135941
***Bambusistroma didymosporum***	**MFLU 15-0057**	**KP761733**	**KP761730**	**KP761737**	**KP761727**
*B. didymosporum*	MFLU 15-0058	KP761734	KP761731	KP761738	KP761728
***Bimuria novae-zelandiae***	**CBS 107.79**	**MH861181**	**AY016356**	**AY016338**	**DQ471087**
***Chromolaenicola nanensis***	**MFLUCC 17-1473**	**MN325015**	**MN325003**	**MN325009**	**MN335648**
*C. nanensis*	MFLUCC 17-1477	MN325014	MN325002	MN325008	MN335647
***C. sapindi***	**KUMCC 21-0564**	**OP058967**	**OP059058**	**OP059009**	**OP135943**
***Cylindroaseptospora leucaenae***	**MFLUCC 17-2424**	**NR_163333**	**NG_066310**	**MK347856**	**MK360047**
***C. siamensis***	**MFLUCC 17-2527**	**MK347760**	**MK347976**	**MK347866**	**MK360048**
***Deniquelata barringtoniae***	**MFLUCC 11-0422**	**NR_111779**	**NG_042696**	**JX254656**	**N/A**
***Dictyoarthrinium vittalii***	**NFCCI4249**	**MF406218**	**MF182395**	**MF622059**	**MF182398**
***D. hydei***	**SQUCC 13296**	**MW077145**	**N/A**	**MW077161**	**MW075771**
***Didymocrea sadasivanii***	**CBS 438.65**	**MH858658**	**DQ384103**	**N/A**	**N/A**
***Didymosphaeria rubi-ulmifolii***	**MFLUCC 14-0023T**	**N/A**	**KJ436586**	**NG_063557**	**N/A**
*D. rubi-ulmifolii*	MFLUCC 14-0024	N/A	KJ436585	KJ436587	N/A
***Kalmusia italica***	**MFLUCC 14-0560**	**KP325440**	**KP325441**	**KP325442**	**N/A**
*K. erioi*	MFLU 18-0832	NR_169984	MN473052	MN473046	MN481599
***Kalmusibambusa triseptata***	**MFLUCC 13-0232**	**KY682697**	**KY682695**	**KY682696**	**N/A**
***Karstenula lancangensis***	**KUMCC 21-0670**	**OP058969**	**OP059060**	**OP059011**	**N/A**
*K. lancangensis*	KUMCC 21-0677	OP058970	OP059061	OP059012	N/A
***Laburnicola hawksworthii***	**MFLUCC 13-0602**	**KU743194**	**KU743195**	**KU743196**	**N/A**
***L. muriformis***	**MFLUCC 14-0921**	**KU743200**	**KU743201**	**KU743202**	**N/A**
*Letendraea cordylinicola*	MFLUCC 11-0150	KM213996	KM213999	KM214002	N/A
***L. cordylinicola***	**MFLUCC 11-0148**	**NR_154118**	**NG_059530**	**KM214001**	**N/A**
*Montagnula donacina*	KUMCC 21-0653	OP058961	OP059052	OP059003	OP135938
***M. thailandica***	**MFLUCC 17-1508**	**MT214352**	**NG070949**	**NG070158**	**MT235774**
***Neokalmusia brevispora***	**KT 1466**	**LC014573**	**AB524600**	**AB524459**	**AB539112**
*N. scabrispora*	KT 1023	LC014575	AB524593	AB524452	AB539106
***Neptunomyces aureus***	**CMG 10A = MUM 19.38**	**MK912119**	**N/A**	**N/A**	**MK948000**
*Neptunomyces* sp.	YNE01044	NMDCN0007RII	NMDCN0007RIV	N/A	NMDCN0007RJ6
***N. chinensis***	**SZU25-020**	**PV730047**	**PV730026**	**PV730037**	**PV749903**
***N. chinensis***	**SZU25-021**	**PV730048**	**PV730027**	**PV730038**	**PV749904**
*N. iteae*	MFLUCC 26-0216	PZ458815	PZ458817	PZ458811	PZ501086
***N. iteae***	**MFLUCC 26-0217**	**PZ458816**	**PZ458818**	**PZ458812**	**PZ501087**
***N. jeanbriggsiae***	**BRIP 75897a**	**PQ431208**	**PQ431200**	**N/A**	**N/A**
***N. juncicola***	**CPC 45436**	**PP791436**	**PP791464**	**N/A**	**PP780627**
***N. litoralis***	**BRIP 75555a**	**OR271911**	**OR259052**	**N/A**	**N/A**
***N. soli***	**MFLUCC 24-0272**	**PP993910**	**PQ306558**	**N/A**	**N/A**
*N. soli*	MFLUCC 24-0279	PQ306559	PQ306560	N/A	N/A
***N. yunnanensis***	**YNE00523**	**NMDCN0007RIH**	**NMDCN0007RIP**	**N/A**	**NMDCN0007RJC**
*Paracamarosporium fagi*	CPC 24890	KR611886	KR611904	N/A	N/A
***P. fagi***	**CPC 24892**	**KR611887**	**KR611905**	**N/A**	**N/A**
***Paramassariaosphaeria anthostomoides***	**MFLUCC 16-0172**	**KU743206**	**KU743207**	**KU743208**	**N/A**
*Paraphaeosphaeria rosae*	MFLUCC 17-2547	MG828935	MG829044	MG829150	MG829222
***P. rosae***	**MFLUCC 17-2549**	**MG828937**	**MG829046**	**MG829152**	**MG829223**
*Phaeodothis winteri*	CBS 182.58	N/A	GU301857	GU296183	N/A
*Pseudocamarosporium propinquum*	MFLUCC 13-0544	KJ747049	KJ813280	KJ819949	N/A
***P. pteleae***	**MFLUCC 17-0724**	**NR_157536**	**MG829061**	**MG829166**	**MG829233**
***Pseudopithomyces entadae***	**MFLUCC 17-0917**	**N/A**	**NG_066305**	**MK347835**	**MK360083**
***P. rosae***	**MFLUCC 15-0035**	**MG828953**	**MG829064**	**MG829168**	**N/A**
***Septofusispora thailandica***	**KUMCC 21-0647**	**OP058971**	**OP059062**	**OP059013**	**OP135945**
*S. thailandica*	KUMCC 21-0652	OP058972	OP059063	OP059014	N/A
***Spegazzinia bromeliacearum***	**URM 8084**	**MK804501**	**MK809513**	**N/A**	**N/A**
*S. camelliae*	WNA03	MZ538526	MZ538560	N/A	MZ567102
***S. camelliae***	**CMU328**	**MH734522**	**MH734521**	**MH734523**	**MH734524**
***Tremateia arundicola***	**MFLU 16-1275**	**KX274241**	**KX274248**	**KX274254**	**KX284706**
***T. guiyangensis***	**GZAAS01**	**KX274240**	**KX274247**	**KX274253**	**KX284705**
***T. murispora***	**GZCC 18-2787**	**NR_165916**	**MK972751**	**MK972750**	**MK986482**
*Verrucoconiothyrium nitidae*	CBS 119209	EU552112	EU552112	N/A	N/A
***Xenocamarosporium acaciae***	**CBS 139895**	**NR_137982**	**NG_058163**	**N/A**	**N/A**
*X. acaciae*	MFLUCC 17-2432	MK347766	MK347983	MK347873	MK360093

Notes: Sequences shaded in blue were isolated in this study. Sequences in bold are the ex-type strains. Unavailable sequences are indicated as “N/A”. The National Microbiology Data Center (NMDC) receives accession numbers for genomic loci that start with “NMDCXXXXXXXX,” while the National Center for Biotechnology Information (NCBI) GenBank receives all other accession numbers.

**Table 4.** The result of best-fit model for each gene region.

**Gene region/loci**	*** Diaporthe ***	*** Didymosphaeriaceae ***
ITS	GTR+I+G	GTR+G
LSU	–	GTR+I+G
SSU	–	GTR+I+G
*tef1-α*	HKY+I+G	GTR+I+G
*tub2*	HKY+I+G	–
*cal*	GTR+I+G	–
*his3*	GTR+I+G	–

## Results

### Phylogenetic Analyses

**Figure 1.**
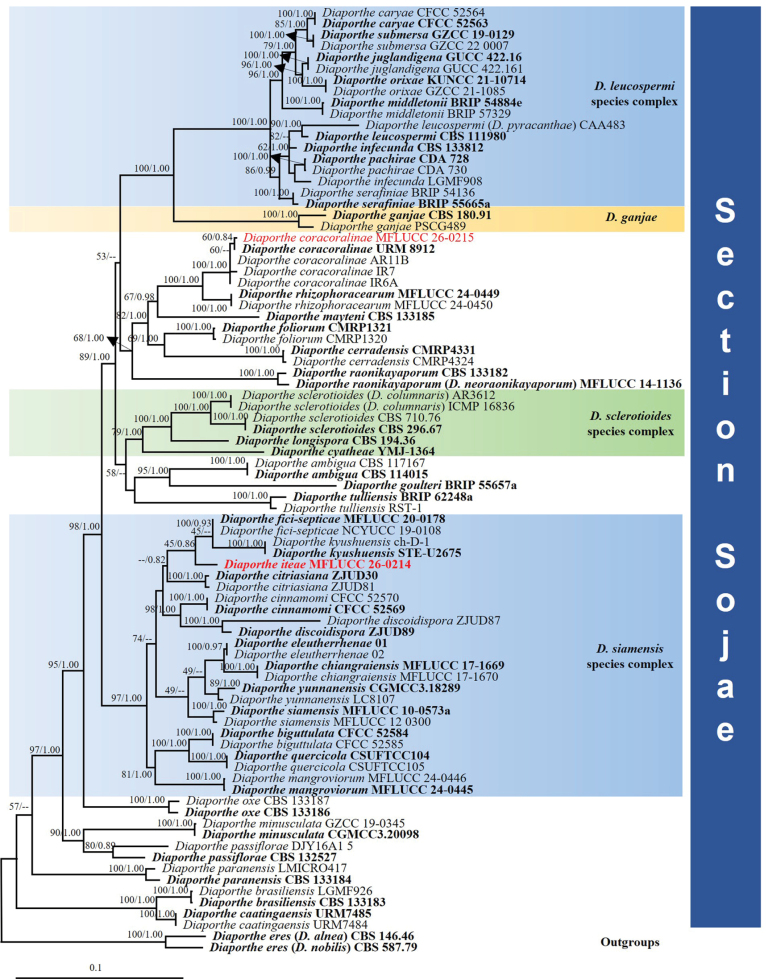
Maximum likelihood phylogram based on combined ITS, *tef1-α*, *tub2*, *cal* and *his3* sequence data. Isolates from present study are in red and ex-type strains are indicated in bold. Bootstrap support values ≥ 40% (left) and Bayesian posterior probabilities ≥ 0.80 (right) are shown at the nodes (ML/BYPP).

Phylogenetic analysis of 84 taxa from *Diaporthe*, section *Sojae* was performed using a concatenated ITS, *tef1-α*, *tub2*, *cal* and *his3* sequence dataset including the outgroup taxa, *D. eres* (= *D. alnea*) CBS 146.46 and *D. eres* (= *D. nobilis*) CBS 587.79 (Fig. 1). The combined alignment dataset contained 2609 total characters (ITS: 1–561 bp, *tef1-α*: 562–904 bp, *tub2*: 905–1672 bp, *cal*: 1673–2151 bp, *his3*: 2152–2609 bp), including gaps. The RAxML analysis generated a best scoring tree with a final ML optimization likelihood value of −23431.396860. The matrix is composed of 1361 distinct alignment patterns, with 29.37% undetermined characters or gaps. Estimated base frequencies were recorded as follows: A = 0.213190, C = 0.326225, G = 0.238836, T = 0.221749; substitution rates AC = 1.227081, AG = 3.737206, AT = 1.377917, CG = 1.142710, CT = 5.072988, and GT = 1.000000. The gamma distribution shape parameter (*α* value) was equal to 0.315160. For Bayesian analysis, the average standard deviation of split frequencies was 0.009337.

**Figure 2.**
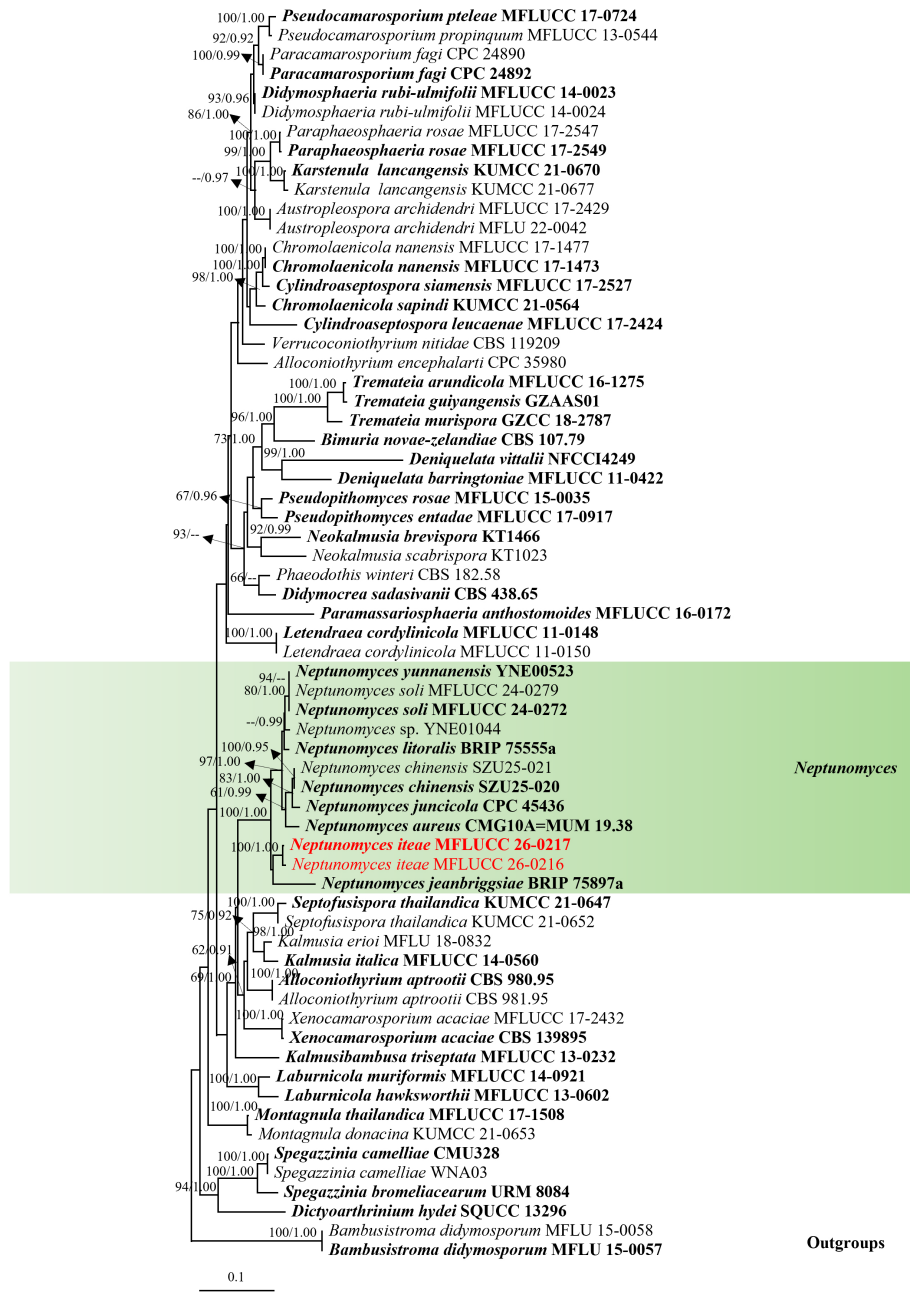
Maximum likelihood phylogram based on combined ITS, LSU, SSU and *tef1-α* sequence data. Isolates from present study are in red and ex-type strains are indicated in bold. Bootstrap support values ≥ 60% (left) and Bayesian posterior probabilities ≥ 0.90 (right) are shown at the nodes (ML/BYPP).

Phylogenetic analysis of 65 taxa in *Didymosphaeriaceae* was performed using a combined ITS, LSU, SSU and *tef1-α* sequence dataset including the outgroup taxa, *Bambusistroma didymosporum*MFLU 15-0057 and *B. didymosporum*MFLU 15-0058 (Fig. 2). The combined alignment dataset contained 3459 total characters (ITS: 1–604 bp, LSU: 605–1506 bp, SSU: 1507–2531 bp, *tef1-α*: 2532–3459 bp), including gaps. The RAxML analysis generated a best scoring tree with a final ML optimization likelihood value of −20608.400551. The matrix is composed of 1131 distinct alignment patterns, with 27.46% undetermined characters or gaps. Estimated base frequencies were recorded as follows: A = 0.238889, C = 0.251162, G = 0.272199, T = 0.237751; substitution rates AC = 1.520570, AG = 2.266778, AT = 1.554952, CG = 1.154130, CT = 6.226753, and GT = 1.000000. The gamma distribution shape parameter (*α* value) was equal to 0.191358. For Bayesian analysis, the average standard deviation of split frequencies was 0.009683.

The phylograms derived from ML and BI analyses were topologically comparable. Thus, the RAxML trees were displayed as backbone trees. The phylogram of *Diaporthe* section *Sojae* revealed that *Diaporthe iteae* sp. nov. (MFLUCC 26-0214) constitutes a distinct sister clade with *D. kyushuensis* (STE-U2675), *D. fici-septicae* (MFLUCC 20-0178) and *D. citriasiana* (ZJUD30) (45% ML, 0.86 BYPP) within *Diaporthe siamensis* species complex (Fig. 1). The new strain MFLUCC 26-0215 clusters with the *D. coracoralinae* (URM8912, ex-type, AR11B, IR7 and IR6A) with high support (100% ML, 1.00 BYPP; Fig. 1). Overall phylogenetic placements of *Diaporthe* section *Sojae* are well-resolved, except *D. cyatheae*, *D. infecunda*, *D. iteae* and *D. mayteni* (Fig. 1).

While the phylogram of *Didymosphaeriaceae* showed that two strains of *Neptunomyces iteae* sp. nov. (MFLUCC 26-0217, ex-type and MFLUCC 26-0216) clusters together with high support (100%ML, 1.00 BYPP; Fig. 2) and closely related with *N. jeanbriggsiae* (BRIP 75897a) (Fig. 2). In addition, many taxa in *Didymosphaeriaceae* (e.g. *Alloconiothyrium aptrootii*, *Cylindroaseptospora leucaenae*, *Didymocrea sadasivanii*, *Kalmusibambusa triseptata*, *Paramassariaosphaeria anthostomoides*, *Phaeodothis winteri*, and *Verrucoconiothyrium nitidae*) constitute poor support lineages (Fig. 2). Particularly, several *Neptunomyces* species (e.g. *Neptunomyces jeanbriggsiae*, *N. litoralis*, *N. soli* and *N. yunnanensis*) occupy unresolved placements (Fig. 2).

### Taxonomy

#### Diaporthe iteae

Taxon classificationFungiDiaporthalesDiaporthaceae

Monkai, Bhat, Hongsanan & S. Lumyong sp. nov.

3A52DDC3-6A65-5559-A1F4-41F10F9BBE3A

Fig. 3

##### Etymology.

Referring to the host genus *Itea* from which the holotype was collected.

##### Holotype.

MFLU 26-0232.

##### Description.

***Endophytic*** on healthy leaves of *Itea japonica*. **Sexual morph**: undetermined. **Asexual morph**: Coelomycetous. ***Conidiomata*** on PDA pycnidial, 500–1800 μm diam., stromatic, semi-immersed, scattered on PDA, subglobose to ovoid, dark brown to black, multilocular with several prominent black necks extending above the stroma, with conidial drops exuding in white to pale-yellow. ***Ostiolar neck*** 800–1400 μm high × 250–400 μm diam., elongated, conical, dark brown to black, papilate. ***Conidiomatal wall*** 80–180 μm diam., comprising multiple layers of brown to reddish brown, arranged in ***textura prismatica*** to ***textura globosa***. ***Conidiophores*** reduced to conidiogenous cells. ***Conidiogenous cells*** 17–38 × 1.5–2.3 μm (x– = 26 × 2 μm, *n* = 10), phialidic, cylindrical, densely aggregated, terminal, slightly tapering towards the apex, hyaline. ***Alpha conidia*** not observed. ***Beta conidia*** 21.8–35.5 × 1–1.8 μm (x– = 28.5 × 1.5 μm, *n* = 30), aseptate, filiform, tapering towards the apex, truncate at the base, sinuous at one end, hyaline, eguttulate, smooth-walled.

**Figure 3.**
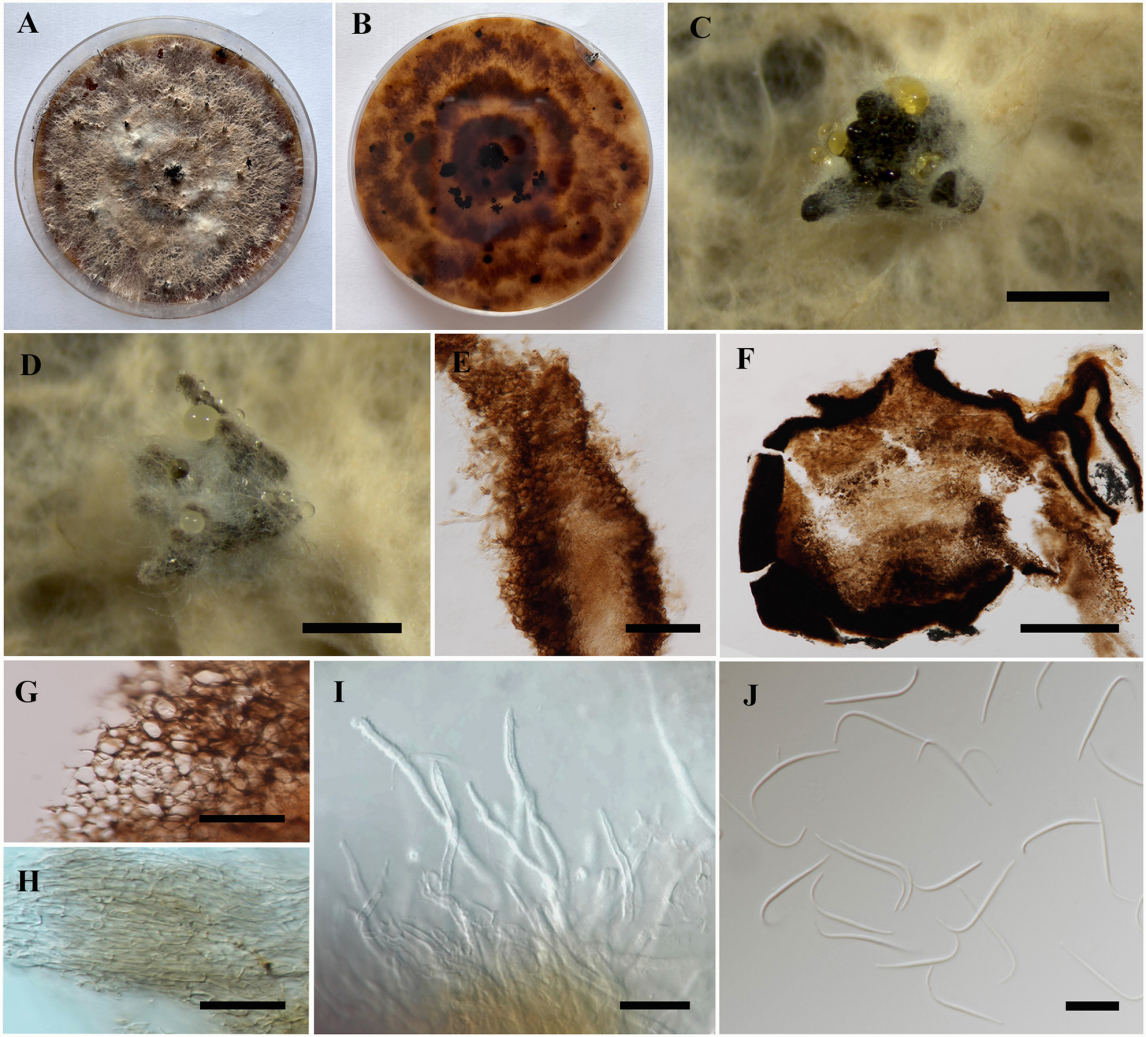
*Diaporthe iteae* (MFLUCC 26-0214, ex-type). **A, B**. Colony after one month on PDA at 28 °C (surface and reverse); **C, D**. Conidiomata and conidial masses; **E**. Ostioles; **F**. Pycnidia; **G, H**. Conidiomata wall; **I**. Conidiogenous cells and conidia; **J**. Conidia. Scale bars: 1000 μm (**C, D**); 200 μm (**E, F**); 10 μm (**G–J**).

##### Culture characteristics.

Colonies on PDA reached at 8 cm diam. in 5 d at 28 °C, raised elevation, effuse, dense aerial mycelium, cottony to floccose, undulate margin, initial white, becoming pale brown due to pigment formation, in reverse, pale brown to reddish brown, sporulated after one month, with white to pale yellowish conidial drops exuding from the ostioles.

##### Material examined.

Thailand • Chiang Mai Province, Hang Dong District, on living leaves of *Itea japonica* (*Iteaceae*), 7 February 2023, J. Monkai, IT26 (MFLU 26-0232, holotype), ex-type living culture, MFLUCC 26-0214 = MBSZU 26-101, dried culture permanently preserved in a metabolically inactive state.

##### Notes.

In the phylogenetic analysis, *Diaporthe iteae* forms a distinct clade and has a close relationship with *D. kyushuensis*, *D. fici-septicae* and *D. citriasiana* (45% ML, 0.86 BYPP; Fig. 1). Morphologically, *D. iteae* resembles *D. fici-septicae* and *D. citriasiana* in having stromatic conidiomata with ostioles (Tennakoon et al. 2021). *Diaporthe iteae* can be distinguished from *D. citriasiana* and *D. kyushuensis* by having shorter beta conidia (21.8–35.5 × 1–1.8 vs. 24–42.5 × 1–2 vs. 25–55 × 1–2 μm) (Kajitani and Kanematsu 2000; Huang et al. 2013). However, *D. fici-septicae* produced only alpha conidia (Tennakoon et al. 2021). Thus, it was unable to compare their conidial morphology. A pairwise nucleotide comparison between *D. iteae* and *D. citriasiana* indicates 3.2%, 5.3%, 3.7%, 6.3%, and 5% bp difference in ITS, *tef1-α*, *tub2*, *cal*, *his3*, respectively (Table 5). *Diaporthe iteae* can be distinguisted from *D. fici-septicae* in 2%, 2.4%, and 2.5% bp difference in ITS, *tef1-α*, *tub2* respectively, and *D. kyushuensis* in 3.4% bp difference in ITS. These substantial nucleotide differences across the selected gene regions (greater than 1.5% bp divergence) further support the distinction of the species, in accordance with the guidelines of Jeewon and Hyde (2016), Chethana et al. (2021) and Maharachchikumbura et al. (2021), which have been widely applied in previous studies (Monkai et al. 2023; Li et al. 2025; Aumentado et al. 2026; Vasighzadeh et al. 2026). Based on its morphological characteristics, phylogenetic analysis and pairwise nucleotide difference, *D. iteae* is described here as a novel species.

**Table 5.** A pairwise nucleotide comparison between *Diaporthe iteae* (MFLUCC 26-0214^T^) and closely related taxa.

**Taxa**	**Gene region/locus**				
	**ITS**	**tef1-α**	**tub2**	**cal**	his3*
*D. citriasiana* (ZJUD30^T^, ZJUD81*)	3.2% (19/592)	5.3% (18/338)	3.7% (20/543)	6.3% (25/399)	5.0% (23/463)
*D. fici-septicae* (MFLUCC 20-0178)	2.0% (10/507)	2.4% (8/336)	2.5% (11/446)	NA	NA
*D. kyushuensis* (STE-U2675^T^)	3.4% (16/465)	NA	NA	NA	NA

Note: The data represented by % base-pair difference (the number of different nucleotides/number of all nucleotides). ^T^ex-type strain, *For *his3* gene region, *D. citriasiana* strain ZJUD81 was used for comparison.

#### Diaporthe coracoralinae

Taxon classificationFungiDiaporthalesDiaporthaceae

L.O. Ferro, Souza-Motta & J.D.P., Bezerra, Fungal Syst. Evol. 14: 259 (2024)

7BB366CC-0B58-5585-804A-EAD4B76670A1

Fig. 4

##### Description.

***Endophytic*** on healthy leaves of *Itea japonica*. **Sexual morph**: undetermined. **Asexual morph**: Coelomycetous. ***Conidiomata*** on PDA pycnidial, 600–3500 μm diam., stromatic, semi-immersed, solitary or aggregated, subglobose to discoid, dark brown to black, multilocular with several locules embedded in the stroma, ostiolate, with conidial drops exuding in white to pale-yellow. ***Ostioles*** up to 200 μm diam., conical, dark brown to black, papilate. ***Conidiomatal wall*** 50–120 μm diam., comprising several layers of brown to dark brown, arranged in ***textura angularis***. ***Conidiophores*** reduced to conidiogenous cells. ***Conidiogenous cells*** 11.5–16 × 1.6–2.4 μm (x– = 14 × 2 μm, *n* = 10), phialidic, cylindrical, densely aggregated, terminal, slightly tapering towards the apex, hyaline. ***Alpha conidia*** not observed. ***Beta conidia*** 22.3–31.5 × 1.2–2 μm (x– = 27 × 1.5 μm, *n* = 30), aseptate, filiform, tapering towards the apex, truncate at the base, slightly curved or sinuous at one end, hyaline, eguttulate, smooth-walled.

**Figure 4.**
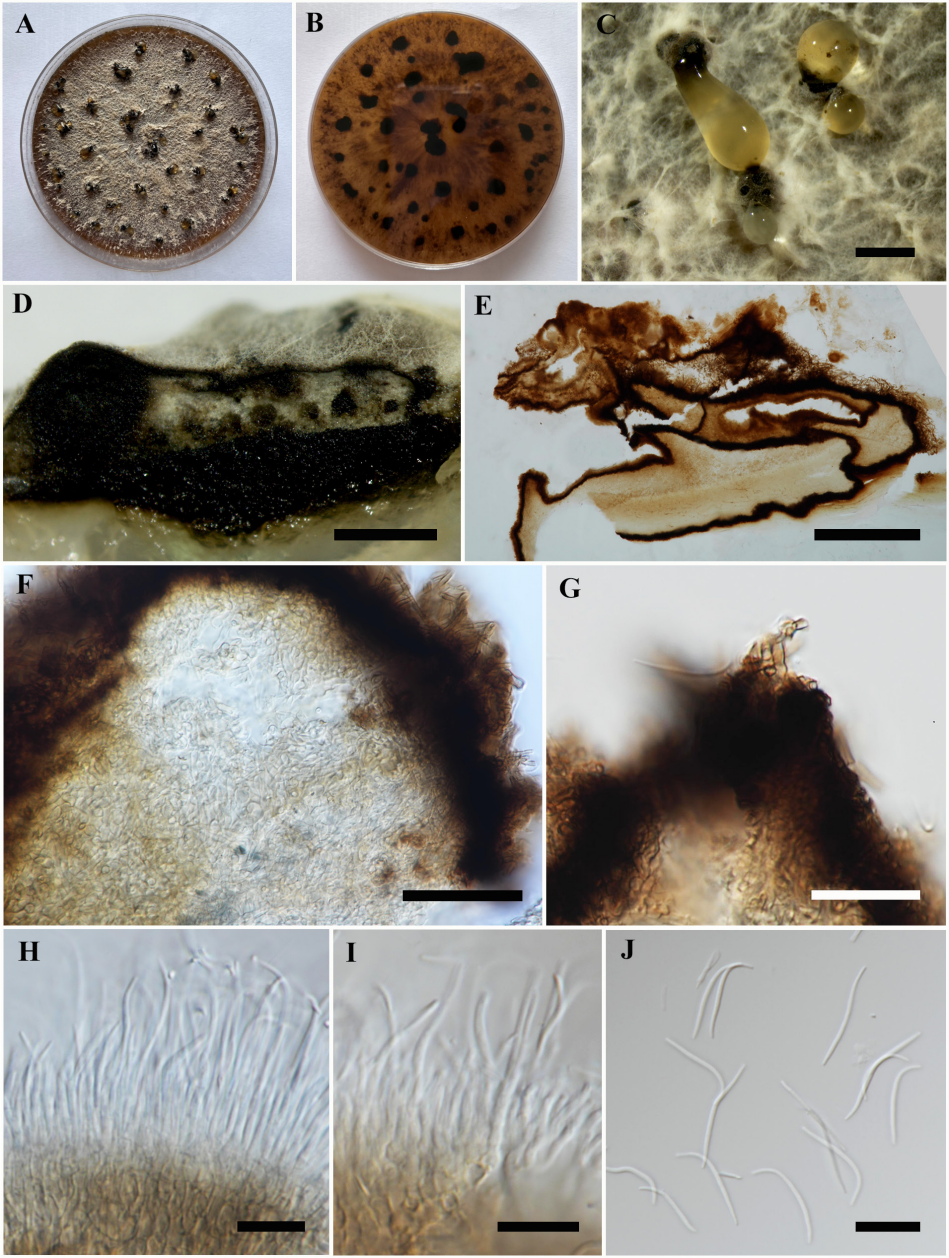
*Diaporthe coracoralinae* (MFLUCC 26-0215). **A, B**. Colony after one month on PDA at 28 °C (surface and reverse); **C**. Conidiomata and conidial masses; **D, E**. Section of conidiomata; **F**. Conidiomata wall; **G**. Ostioles; **H, I**. Conidiogenous cells and conidia; **J**. Conidia. Scale bars: 1000 μm (**C**); 500 μm (**D, E**); 100 μm (**F, G**); 10 μm (**H, I**).

##### Culture characteristics.

Colonies on PDA reached at 8 cm diam. in 5 d at 28 °C, flat elevation, effuse, sparse aerial mycelium, floccose to strongly rugose, filiform margin, initial white, becoming pale brown due to pigment formation, in reverse, pale brown to dark brown, sporulated after one month, with white to pale yellowish conidial drops exuding from the ostioles.

##### Material examined.

Thailand • Chiang Mai Province, Hang Dong District, on living leaves of *Itea japonica* (*Iteaceae*), 7 February 2023, J. Monkai, IT27 (MFLU 26-0233), living culture, MFLUCC 26-0215 = MBSZU 26-102, dried culture permanently preserved in a metabolically inactive state.

##### Notes.

*Diaporthe coracoralinae* was described as an endophyte on healthy branches of *Brosimum gaudichaudii* from Brazil (Ferro et al. 2024). In our phylogenetic analysis, strain MFLUCC 26-0215, isolated from leaves of *Itea japonica* from Thailand, clusters with the type strain (URM8912) and other representative strains of *D. coracoralinae* with strong statistical support (100% ML, 1.00 BYPP; Fig. 1). A pairwise nucleotide comparison of ITS, *tef1-α*, *tub2*, *cal* and *his3* between our collection and the type strain of *D. coracoralinae* (URM8912) indicates that they are identical, except for a 2 bp difference (0.5%) in *his3* (Ferro et al. 2024). Ferro et al. (2024) reported that this species produce hyaline, aseptate, fusiform alpha conidia (5–7 × 2–3 μm). Whereas, beta and gamma conidia were not observed. Our study first time reports the production of beta conidia on *D. coracoralinae* which are hyaline, aseptate, filiform and eguttulate. Hence, we identify the new collection as *D. coracoralinae*, marking a new host record on *Itea* and a new geographical record from Thailand.

#### Neptunomyces iteae

Taxon classificationFungiPleosporalesDidymosphaeriaceae

Monkai, Bhat, Hongsanan & S. Lumyong sp. nov.

C00C669C-E6AF-552F-A40F-2121AAFF4AE0

Fig. 5

##### Etymology.

Referring to the host genus *Itea* from which the holotype was collected.

##### Holotype.

MFLU 26-0235.

##### Description.

***Endophytic*** on healthy stems and leaves of *Itea riparia*. **Sexual morph**: undetermined. **Asexual morph**: Coelomycetous. ***Conidiomata*** on PDA pycnidial, 200–800 μm diam., erumpent, immersed to semi-immersed, covered by dense mycelium, globose to subglobose, dark brown to black, solitary to gregarious, releasing black conidial masses. ***Conidiomatal wall*** 22–58 μm wide (x– = 41 μm), comprising a few layers of brown to dark brown pseudoparenchymatous cells, arranged in ***textura angularis***. ***Conidiophores*** 1.7–4 μm diam., aggregated in clusters on hyphae, smooth, septate, branched, straight or flexuous, hyaline to pale brown. ***Conidiogenous cells*** 5–15.5 × 1.5–3 μm (x– = 8 × 2.3 μm, *n* = 10), holoblastic, discrete, subcylindrical to clavate, hyaline, smooth. ***Conidia*** 4.8–7 × 3–4.3 μm (x– = 6 × 3.7 μm, *n* = 30), aseptate, ellipsoidal to limoniform, apex acute to apiculate, widest in the middle, tapering towards a narrowly truncate base, brown to golden brown, guttulate, smooth, thick-walled.

**Figure 5.**
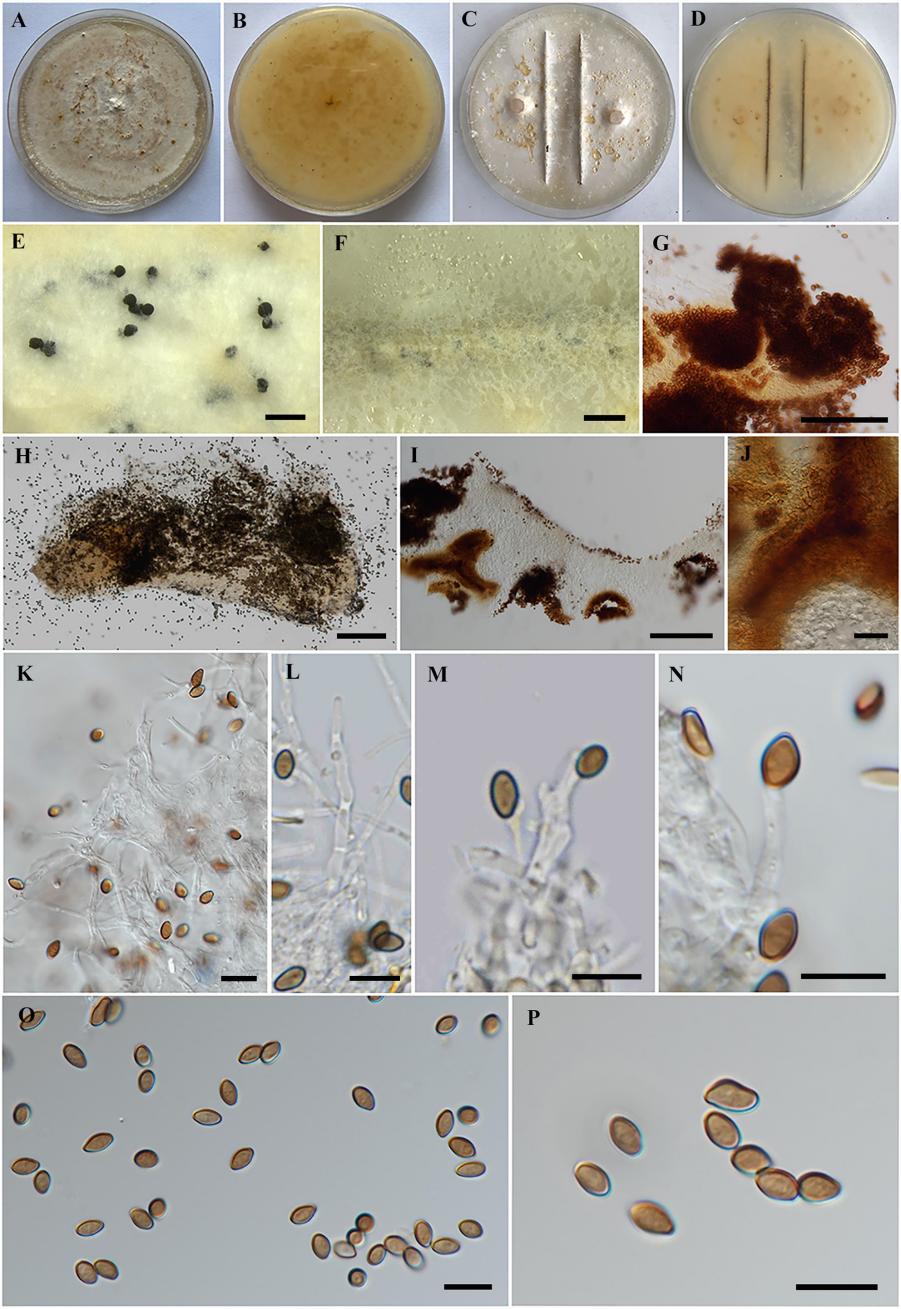
*Neptunomyces iteae* [MFLUCC 26-0217, ex-type (**A, B**), MFLUCC 26-0216 (**C, D**)]. **A–D**. Colony after one month on PDA at 28 °C (surface and reverse); **E, F**. Conidiomata and conidial masses on toothpick and PDA; **G, H**. Pycnidia and conidiomata wall; **I–K**. Conidiophores, conidiogenous cells and conidia; **L, M**. Conidia. Scale bars: 500 μm (**E, F**); 200 μm (**G–I**); 10 μm (**J–P**).

##### Culture characteristics.

Colonies on PDA reached at 5 cm diam. in 7 d at 28 °C, raised elevation, cottony, entire margin, initial white, becoming yellowish-white with age, slightly immersed into agar, in reverse, whitish to yellowish in the middle, sporulated after one to two months, with numerous black conidial masses.

##### Material examined.

Thailand • Chiang Mai Province, Mae Rim District, on living stems of *Itea riparia* (*Iteaceae*), 10 July 2023, J. Monkai, IT82 (MFLU 26-0235, holotype), ex-type living culture, MFLUCC 26-0217 = MBSZU 26-103, dried culture permanently preserved in a metabolically inactive state; • ibid., on living leaves of *Itea riparia* (*Iteaceae*), 10 July 2023, J. Monkai, IT60 (MFLU 26-0234), living culture, MFLUCC 26-0216 = MBSZU 26-104, dried culture permanently preserved in a metabolically inactive state.

##### Notes.

Based on multiloci phylogeny, *Neptunomyces iteae* constitutes an independent lineage sister to *N. jeanbriggsiae* (Fig. 2). Besides, two new isolates cluster together with robust statistically support (100% ML, 1.00 BYPP; Fig. 2). A pairwise nucleotide comparison between *N. iteae* (MFLUCC 26-0217) and *N. jeanbriggsiae* (BRIP 75897a) indicates 9.9% (58/587) bp difference in ITS and 1% (9/870) bp difference in LSU, which further distinguishes the two species. The morphological description of *N. jeanbriggsiae* was not provided (Tan et al. 2024), thus it is not possible to compare its morphological characteristics with our new species. *Neptunomyces iteae* has morphological affinities with *N. chinensis*, *N. soli* and *N. yunnanensis* in having immersed to semi-immersed, globose to subglobose conidiomata and golden yellow to golden brown, ellipsoidal to limoniform conidia with an acute to apiculate apex (Yasanthika et al. 2024; Chen et al. 2025; Tennakoon et al. 2025) (Table 6). However, *N. iteae* is distinct from those species by the presence of conidiophores and longer conidiogenous cells (5–15.5 × 1.5–3 μm in *N. iteae* vs. 4–7 × 3–4 μm in *N. chinensis* vs. 3–7 × 2.5–7 μm in *N. soli* vs. 4.5–6 × 3.9–4.2 μm in *N. yunnanensis*) (Yasanthika et al. 2024; Chen et al. 2025; Tennakoon et al. 2025) (Table 6). Therefore, *N. iteae* is proposed as a new species based on its morphology, phylogenetic placement and pairwise nucleotide difference.

**Table 6.** Synopsis of *Neptunomyces* species. The new species is indicated in bold.

***Neptunomyces* species**	**Hosts and habitats**	**Countries**	**Sexual morphological characters**	**Asexual morphological characters**				**References**
				**Conidiomata**	**Conidiophores**	**Conidiogenous cells**	**Conidia**	
*N. aureus*	*Gracilaria gracilis*	Portugal	UD	Immersed or rarely superficial, dark brown, globose to subglobose, aggregated or solitary	Reduced to conidiogenous cells	5.2 ± 0.3 × 2.0 ± 0.6 μm, hyaline, ampulliform to subcylindrical	7.0 ± 0.6 × 2.7 ± 0.2 μm, aseptate, golden yellow, subcylindrical with rounded apices	Gonçalves et al. (2019)
*N. chinensis*	Dead leaves of *Phragmites australis*	China (Guangdong Province)	UD	90–110 × 80–150 μm, pycnidial, immersed to semi-immersed, dark brown to black, globose to sub-globose, solitary to aggregated	Reduced to conidiogenous cells	4–7 × 3–4 μm, holoblastic, discrete, hyaline, globose to doliiform	6.5–8 × 4–5 μm, aseptate, hyaline to pale brown, ellipsoidal to limoniform, apex acute to apiculate	Tennakoon et al. (2025)
***N. iteae***	**Healthy stems and leaves of *Itea riparia***	**Thailand**	**UD**	**200–800 μm diam., pycnidial, immersed to semi-immersed, dark brown to black, globose to subglobose, solitary to gregarious**	**1.7–4 μm diam., septate, branched, hyaline to pale brown**	**5–15.5 × 1.5–3 μm, holoblastic, discrete, hyaline, subcylindrical to clavate**	**4.8–7 × 3–4.3 μm, aseptate, brown to golden brown, ellipsoidal to limoniform, apex acute to apiculate**	**This study**
*N. jeanbriggsiae*	Unidentified pink basidiocarp on the trunk of an unidentified tree	Australia	UD	UD	UD	UD	UD	Tan et al. (2024)
*N. juncicola*	Culms of *Juncus maritimus*	Netherlands	Pseudothecial, subglobose, papillate, asci fusoid-ellipsoid, ascospores tri-to multiseriate, fusoid-ellipsoid, 5–7 transverse septa, golden brown	180–220 μm diam., pycnidial, ostiolate, brown, globose	3–5 × 4–5 μm, phialidic, hyaline, ampulliform	UD	7–8 × 2–2.5 μm, aseptate, golden brown, subcylindrical to fusoid-ellipsoid, apex subobtuse, hilum truncate	Crous et al. (2024)
*N. litoralis*	Sand	Australia	UD	UD	UD	UD	UD	Tan et al. (2023)
*N. soli*	Soil	Thailand	UD	Brown to dark brown, globose to subglobose, superficial to semi-immersed	UD	3–7 × 2.5–7 μm, hyaline to subhyaline, doliiform to obovoid	5–6.5 × 3–5 μm, aseptate, hyaline to golden yellow, ellipsoidal to fusiform	Yasanthika et al. (2024)
*N. yunnanensis*	Healthy leaves of *Oryza meyeriana* subsp. *granulata*	China (Yunnan Province)	UD	Up to 400 μm diam., pycnidial, immersed, dark brown to black, subglobose, solitary or aggregated	UD	4.5–6 × 3.9–4.2 μm, phialidic, hyaline, ampulliform	3.9–6 × 2.6–3.9 μm, aseptate, golden brown, ellipsoid to fusoid	Chen et al. (2025)

## Discussion

The present study adopts a dual approach including morphological comparison, multiloci phylogenetic construction coupled with nucleotide polymorphism analyses to characterize endophytic coelomycetous fungi associated with *Itea* from Thailand. As a result, two new species are described: *Diaporthe iteae* from *Itea japonica* and *Neptunomyces iteae* from *I. riparia*. Additionally, we report the first record of *D. coracoralinae* from *Itea* in Thailand, which was previously isolated from *Brosimum* in Brazil.

Taxonomic circumscription of *Diaporthe* has earlier been extremely ambiguous in view of intraspecific variability and phenotypic plasticity among species (Dissanayake et al. 2017). The concept of species complex was proposed by Norphanphoun et al. (2022) which delineated *Diaporthe* into 13 species complexes based on phylogenetic analyses of individual and combined five loci (ITS, *tef1-α*, *tub2*, *cal* and *his3*) sequence datasets. Aligning with this phylogenetic reevaluation, Hongsanan et al. (2023) offered a comprehensive review of recognized or suspected *Diaporthe* species and proposed taxonomic modifications for select species based on updated and robust information. Subsequently, Dissanayake et al. (2024) applied a sectional framework to reassess *Diaporthe* species into seven phylogenetically distinct sections including 15 species-complexes and 13 species using integrative phylogenetic approaches. Our study follows the framework of Dissanayake et al. (2024) by applying this concatenated five loci phylogeny, the placements of two species (*D. iteae* and *D. coracoralinae*) belong to section *Sojae* (Fig. 1). Within the section, a new species, *D. iteae* is placed under the *Diaporthe siamensis* species complex and together they form distinct lineage sister to *D. kyushuensis*, *D. fici-septicae* and *D. citriasiana* (Fig. 1). Whereas strain MFLUCC 26-0215 exhibits a close affinity with *D. coracoralinae* and the lineage occupies outside the refined species-complexes (Fig. 1). Notably, the clade comprises *D. cerradensis*, *D. coracoralinae*, *D. mayteni*, *D. foliorum*, *D. raonikayaporum* and *D. rhizophoracearum* is closely related with the *D. ganjae* species complex and the *D. sclerotioides* species complex (Fig. 1); thus, it could be introduced as a novel species-complex by further accurate systematic framework. Moreover, we indicate rather poor support for the branch of *D. iteae* (45% ML, 0.86 BYPP; Fig. 1). This is probably due to the lack of *cal* and *his3* sequence data in *D. fici-septicae* and the lack of *tef1-α*, *tub2*, *cal* and *his3* sequence data in *D. kyushuensis* (Table 5). Incomplete sequence data, particularly *tub2*, *cal* and *his3*, have been found in many *Diaporthe* species (Norphanphoun et al. 2022; Dissanayake et al. 2024). These protein-coding loci are now considered to be effective phylogenetic markers for species delineation (Norphanphoun et al. 2022; Monkai et al. 2023; Dissanayake et al. 2024; Li et al. 2025; Vasighzadeh et al. 2026). Consequently, additional sequence data from both existing and new collections are necessitated to resolve uncertain placements of *Diaporthe* species.

*Neptunomyces* is an ecologically diverse genus that occupies both marine and terrestrial niches (Gonçalves et al. 2019; Tennakoon et al. 2025). The type species, *N. aureus* was originally discovered as an algicolous fungus (epiphytic or endophytic) in Portugal (Gonçalves et al. 2019). The ecological distribution of this genus has been expanded to include soil, sand, and a variety of terrestrial plants in which it serves as endophytes and saprobes (Gonçalves et al. 2019; Tan et al. 2023, 2024; Crous et al. 2024; Yasanthika et al. 2024; Chen et al. 2025; Tennakoon et al. 2025). In this study, we introduce *Neptunomyces iteae* as an endophyte of *Itea riparia* in Thailand. Our discovery, together with existing records from Australia, China, Thailand and the Netherlands, points to a cosmopolitan distribution for the genus, covering temperate and tropical climates across three continents (Gonçalves et al. 2019; Tan et al. 2023, 2024; Crous et al. 2024; Yasanthika et al. 2024; Chen et al. 2025; Tennakoon et al. 2025).

Morphologically, *Neptunomyces* is typically classified by its asexual morph with globose to subglobose, immersed to semi-immersed, pycnidial conidiomata, holoblastic or phialidic conidiogenous cells and ellipsoid or fusiform to limoniform, initially hyaline, later golden yellow to golden brown conidia with round apices (Gonçalves et al. 2019; Crous et al. 2024; Yasanthika et al. 2024; Chen et al. 2025; Tennakoon et al. 2025) (Table 6). Notably, *N. iteae* produces conidiophores, a character not rarely observed in other species. This finding contributes an important dimension to the generic description. Sexual morphs are still uncommon as they have only been recorded in *N. juncicola* (Crous et al. 2024). The absence of morphological data for *N. jeanbriggsiae* and *N. litoralis* (Tan et al. 2023, 2024), emphasizes the necessity of employing molecular data to delineate species.

Previous studies have applied various combinations of ribosomal DNA regions (e.g. ITS, LSU and/or SSU) and protein-coding loci (e.g. *tef1-α*, *tub2* and/or *rpb2*) for the phylogenetic reconstruction of taxa within *Didymosphaeriaceae* (Gonçalves et al. 2019; Ren et al. 2022; Wanasinghe et al. 2024; Chen et al. 2025; Tennakoon et al. 2025). In accordance with Tennakoon et al. (2025) and based on available reference sequence data, we utilized a four-loci dataset of ITS, LSU, SSU and *tef1-α* to perform the phylogenetic analyses of *Neptunomyces*. However, our phylogenetic tree demonstrates several unresolved positions (Fig. 2). *Neptunomyces iteae* clusters with *N. jeanbriggsiae* with low support, forming a branch significantly distant from the well-supported core clade (97% ML, 1.00 BYPP, Fig. 2). In addition, we observe that *N. yunnanensis* and *N. soli* share a close relationship with high support (80% ML, 1.00 BYPP, Fig. 2). A pairwise nucleotide comparison of ITS and LSU between the type strains of *N. yunnanensis* and *N. soli* indicates that they are identical and conspecific (Yasanthika et al. 2024; Chen et al. 2025). However, *tef1-α* sequence data for *N. soli* are lacking (Yasanthika et al. 2024). Morphologically, *N. yunnanensis* resembles *N. soli* in having phialidic, hyaline, doliiform to ampulliform conidiogenous cells with overlapping size ranges (4.5–6 × 3.9–4.2 vs. 3–7 × 2.5–7 μm) and golden yellow to golden brown, ellipsoidal to fusiform conidia with the same size (3.9–6 × 2.6–3.9 vs. 5–6.5 × 3–5 μm) (Yasanthika et al. 2024; Chen et al. 2025). We recommend treating *N. yunnanensis* as a synonym of *N. soli* until *tef1-α* sequence data are acquired and verified. Unstable placements and weak support for these taxa are likely caused by missing sequence data. The lack of SSU and *tef1-α* sequences across all type strains limits the resolution of the *Didymosphaeriaceae* backbone tree (Wanasinghe et al. 2016, 2024; Gonçalves et al. 2019; Tennakoon et al. 2025). Future investigations need to focus on protein-coding markers (e.g. *tef1-α*, *tub2*, *rpb2*) and extensive sampling to clarify species identification and establish a resolved and stable phylogenetic framework for taxa in *Didymosphaeriaceae*.

Taxonomic investigation into the endophytic fungal diversity associated with *Itea* in Northern Thailand has revealed a significant and previously underestimated level of species richness. Our ongoing studies have successfully isolated several noteworthy taxa from *Itea japonica* and *I. riparia* in Thailand including the discovery of three new species (i.e. *Neopestalotiopsis iteae*, *Nigrospora iteae* and *Pseudopestalotiopsis iteae*) alongside four new host records from *Itea* (i.e. *Apiospora vietnamensis*, *Neopestalotiopsis chrysea*, *N. haikouensis*, and *Pestalotiopsis jinchanghensis* (Monkai et al. 2025, 2026). This work considerably advances our knowledge of fungal communities associated with these plant species. Hence, future research is essential to expand sampling efforts throughout various geographical regions in Thailand and China to explore additional fungal lineages. Furthermore, examining the pathogenic potential of these isolates is crucial to characterize disease dynamics and developing sustainable management plans for the conservation of medicinal plant hosts and forest resources within this ecologically significant area.

## Supplementary Material

XML Treatment for Diaporthe iteae

XML Treatment for Diaporthe coracoralinae

XML Treatment for Neptunomyces iteae

## Additional information

### Artificial Intelligence (AI) use

The authors accept full responsibility for the content of the manuscript, including the disclosure of any use of AI.

No AI tools were used in the preparation of this manuscript.

### Author ORCIDs

J. Monkai https://orcid.org/0000-0001-6043-0625

R. Phookamsak https://orcid.org/0000-0002-6321-8416

S. Khuna https://orcid.org/0000-0003-1999-4001

D.J. Bhat https://orcid.org/0000-0002-3800-5910

T. Swe Zin Ei https://orcid.org/0009-0005-9585-8863

J. Xu https://orcid.org/0000-0002-2485-2254

S. Hongsanan https://orcid.org/0000-0003-0550-3152

S. Lumyong https://orcid.org/0000-0002-6485-414X

### Data availability

All of the data that support the findings of this study are available in the main text. The concatenated alignments and phylogenetic trees are available in Figshare (https://doi.org/10.6084/m9.figshare.33091040).
